# Left Atrial Stiffness, a Marker of Atrial Cardiomyopathy, and Atrial Fibrillation - Relationships and Predictors for Procedure Success after Catheter Ablation

**DOI:** 10.5935/abc.20190087

**Published:** 2019-05

**Authors:** Tan Chen Wu

**Affiliations:** Instituto do Coração - Faculdade de Medicina da Universidade de São Paulo, São Paulo, SP - Brazil

**Keywords:** Atrial Fibrillation, Catheter Ablation, Atrial Function, Left, Atrial Remodeling, Recurrence, Treatment Outcome

Over the past years, catheter ablation (CA) for atrial fibrillation (AF) has established itself as a well-recognized strategy in the management of patients with AF and an important option for rhythm control. Although CA is more effective than antiarrhythmic drug therapy, AF recurrences are common during the follow-up.^1^

Late recurrence, during the first 9 months after the blanking period, occurs in 25%-40% of cases and is predominantly linked to the recovery of electrical conduction between the pulmonary veins (PVs) and the left atrium (LA), irrespective of the type of AF. The incidence of very late recurrence (after more than 12 months postablation) has been shown to be higher than previously expected, with an annual recurrence rate estimated at 7.6%.^2^ Bunch et al.^3^ reported AF recurrence rates ranging from 52% (≤ 50 years + paroxysmal AF) to 75% (> 80 years + paroxysmal AF).^3^ In a series of 509 consecutive patients undergoing paroxysmal AF ablation by Teunissen et al., after a single procedure, antiarrhythmic drugs free success rate was 41.3%^4^. The predominant mechanism of very late recurrence includes, in addition to the PV connection, the development of non-PV triggers, and development and maturation of substrate. The predictors appears to be the nonparoxysmal form of AF at baseline, organic heart disease, advanced age, and obesity.

AF is often associated with atrial structural remodeling and causes LA fibrosis/scarring and dilatation. Substrate progression is a multifactorial and time-dependent response of cardiac myocytes to varying "stressors", including electrical, mechanical, and metabolic stressors. Some components of the LA changes are reversible (adaptive), whereas others are permanent (maladaptive). Most risk factors affect AF by causing structural remodeling. Progression of atrial damage due to underlying heart disease is a major factor. Recent studies suggest that AF recurrence can be prevented by effectively managing risk factors such as sleep apnea, obesity, high blood pressure, hyperglycemia, and dyslipidemia, presumably by curtailing further damage and/or reversing existing abnormalities. Conversely, AF itself can cause progression of the substrate. In addition to complexion-channel remodeling that accelerates repolarization and alters conduction properties, rapid activation of atrial cardiomyocytes causes profibrotic changes in fibroblast function and promotes atria fibrosis.

Increased LA scar is associated with increases LA stiffness, which reflects a deteriorated reservoir function. Therefore, LA stiffness could be associated with LA histological changes and predicts sinus rhythm maintenance after treatment in AF patients.^5^ Timely intervention for patients with these conditions may interrupt and perhaps reverse LA remodeling, with a consequent reduction in LA size and improved function.

The scar tissue formation after CA may also adversely impact the diastolic properties of the LA, especially after multiple ablation procedures, worsening the diastolic function or LA compliance. Stiff LA syndrome has been recognized as pulmonary hypertension and dyspnea that develops after CA, a potential complication of the procedure with a low prevalence.^6,7^

Thus, evaluation of the LA as cardiovascular biomarker, especially in AF, has become increasingly important.^8,9^ LA remodeling is monitored in clinical practice using various noninvasive imaging modalities, but it has not been yet incorporated into clinical decision making. In this published issue, Correia et al.,^10^ investigated, through a systematic review and meta-analysis, if LA stiffness could be a predictor of AF recurrence after CA, and to discuss its clinical use.^10^ Only 4 prospective observational studies were included in the systematic review and 3 of them in the meta-analysis, with different methods, and most of all used LA pressure measured invasively during CA to estimate LA stiffness. They found that LA stiffness was a strong independent predictor of AF recurrence after CA (HR = 3.55, 95% CI 1.75-4.73, p = 0.0002), and concluded that a non-invasive assessment of LA stiffness prior to CA can be used as a potential screening factor to select or to closely follow patients with higher risks of AF recurrence and development of the stiff LA syndrome. The small number of studies, with heterogeneity and a short mean follow-up in 3 studies were limitations in this meta-analysis.

These findings add to our knowledge by clarifying the association between atrial remodeling and outcomes after AF ablation. Current guidelines recommendation is to perform CA as second-line treatment after failure or intolerance to at least one antiarrhythmic drug. As first-line treatment, the indication recommendations are weaker and only limited to patients with paroxysmal AF. These recommendations usually lead physicians to treat patients with CA after a longer history period of clinical AF. The development of tools and methods to determine markers of atrial cardiomyopathy may allow to avoid the mismatch of the best time for CA, in accordance with more substrate and patient-oriented process of diagnosis and therapy of AF. Certainly, further studies will be required to support identification by noninvasive cardiac imaging of patients for whom CA should be considered early before there is significant LA functional remodeling with associated fibrosis.
